# The Value of Four-Quadrant Cervical Biopsy in Women with Different Colposcopic Impressions

**DOI:** 10.3390/diagnostics13142384

**Published:** 2023-07-16

**Authors:** Mandy Man-Yee Chu, Charleen Sze-Yan Cheung, Siew-Fei Ngu, Ka-Yu Tse, Philip Pun-Ching Ip, Annie Nga-Yin Cheung, Hextan Yuen-Sheung Ngan, Karen Kar-Loen Chan

**Affiliations:** 1 Department of Obstetrics and Gynecology, The University of Hong Kong, Queen Mary Hospital, Hong Kong; 2 Department of Pathology, The University of Hong Kong, Queen Mary Hospital, Hong Kong

**Keywords:** cervical intra-epithelial neoplasia, random cervical biopsy, colposcopy

## Abstract

The aim of this study was to compare the diagnostic efficacy of colposcopic-directed biopsy and four-quadrant biopsy in detecting high-grade cervical intra-epithelial neoplasia (CIN). Women attending three women’s clinics for routine cervical screening were recruited. Colposcopy was arranged for women with any cytologic abnormalities greater than atypical squamous cells of undetermined significance (ASCUS), two consecutive ASCUS results or positive HPV testing. During colposcopy, a cervical biopsy was taken from the most suspicious area, but more than one biopsy was allowed. Four-quadrant biopsies at 3, 6, 9 and 12 o’clock and an endocervical curettage were also taken in all cases. A total of 1522 colposcopies were performed in 1311 subjects from June 2010 to August 2017, with 118 cases of high-grade CIN diagnosed. Colposcopic-directed biopsy detected 50.8% of the 118 high-grade CIN, while four-quadrant biopsy detected 86.4% (*p* < 0.0001). Twenty-seven cases (22.9%) of high-grade CIN were diagnosed in women with normal or unsatisfactory colposcopy. Among the 64 cases with low-grade colposcopic impression, four-quadrant biopsy detected significantly more high-grade CIN (53 cases, 82.8%) than colposcopic-directed biopsy (35 cases, 56.3%) (*p* = 0.0011). Four-quadrant cervical biopsies should be considered for all women with an abnormal smear or positive HPV testing, especially in patients with low-grade/normal/unsatisfactory colposcopy.

## 1. Introduction

Cervical cancer is the fourth most common cancer in women, with an estimated 604,000 new cases and 342,000 deaths in the world in 2020. It is one of the most preventable malignancies through well-organized screening programs that aim at detecting and treating pre-malignant lesions before they progress to invasive disease. The success of a screening program depends not only on the coverage rate of the targeted population and the quality of the samples and laboratory tests but also the appropriate management of women with abnormal screening results. 

For more than half a century, colposcopy and directed biopsy have been regarded as the most important investigations in the management of women with abnormal screening results. Different professional bodies have developed clinical guidelines to guide the standard of the provision of colposcopy services [1,2,3]. However, the practice of colposcopy varies greatly between colposcopists and in different parts of the world. A recent retrospective study showed that most colposcopists in US community-based clinics performed less than three colposcopies a month, and the colposcopic impression was only documented in 41% of the cases. The overall sensitivity of colposcopy impression in predicting histology-confirmed high-grade CIN was only 36.5% in this study [4]. Training and accreditation programs are available in some parts of the world to improve the competence of colposcopists. Despite all these efforts, a few recent studies showed that the sensitivity of colposcopy in detecting high-grade CIN was as low as 30–60% [5,6], and it seemed that the accuracy of colposcopy was not related to the training background or experience [7,8]. In view of this, in the past decade, there has been increasing interest in the role of random cervical biopsy during colposcopy. Studies have shown that random biopsy might improve the sensitivity of detecting high-grade cervical intra-epithelial neoplasia (CIN) in women in different clinical settings [9,10,11,12].

The aim of this study is to compare the diagnostic efficacy of colposcopic-directed biopsy and four-quadrant random biopsy in detecting high-grade CIN in a Chinese population in the setting of a clinical study. 

## 2. Materials and Methods

This study was part of a population-based, randomized controlled study on the effect of HPV-cytology co-testing as primary screening strategy in Chinese population. Chinese women, aged 30–60 years old, attending three women’s health clinics in Hong Kong for routine cervical cancer screening were recruited to participate in the study. Eligible women were given an information sheet containing details on cervical smears, HPV testing and the present study, and all women provided written consent before any study procedures. 

The details of the study design, methods and the result of the effectiveness of HPV-cytology co-testing have been previously published [13]. In short, each consented participant was randomized on a 1:1 ratio into one of two arms: cytology arm (control) or cytology plus HPV testing arm (intervention). In both groups, colposcopy would be offered to women with any cytologic abnormalities greater than ASCUS or two consecutive ASCUS results. In the control group, colposcopy would also be arranged for women with ASCUS smear and positive HPV testing. In the intervention group, women with positive HPV testing, regardless of the smear results, would also be referred for colposcopy. 

All the colposcopy examinations were performed by 13 accredited colposcopists, each with an experience of at least 150 colposcopies, at two government hospitals. The colposcopists were blinded to the group allocation and the cytology and HPV results. During colposcopy, the cervix was first cleaned with normal saline and inspected under the colposcope. Acetic acid was then applied to the cervix, and the presence of acetowhite areas, as well as abnormal vascular patterns, was noted. Lugol’s iodine was also applied as an adjunct for the colposcopy examination. The colposcopist documented the findings in a standard proforma, which included the presence of acetowhite lesion and its details, any coarse punctations or mosaicism. The colposcopists then drew the lesions on the diagram, gave a colposcopic impression and stated the site where the colposcopic-directed biopsies were taken. Cervical biopsy was then taken from the most suspicious area, but more than one biopsy site was allowed in patients with multi-focal lesions, and four-quadrant biopsies at 3, 6, 9 and 12 o’clock and an endocervical curettage was also taken in all cases. If the suspicious area fell into one of the four-quadrant biopsy sites, the result was marked as both colposcopic-directed biopsy and 4-quadrant cervical biopsy at that site. The cervical biopsies were taken using Belinson cervical biopsy forceps and sent for pathological examination in separate vials.

All the cervical biopsies were embedded separately, i.e., one piece of biopsy tissue in one formalin-fixed, paraffin-embedded block. Deeper sections and p16 immunohistochemistry were performed as indicated to obtain a definitive diagnosis of high-grade CIN, adenocarcinoma in situ or invasive lesions.

Statistical analyses were conducted using SPSS version 26.0 for Windows. The comparison between the two methods was calculated using chi-square test. A *p*-value ≤ 0.05 was considered statistically significant. 

This trial was registered at ClinicalTrials.gov accessed on 9 May 2023 (NCT01058460) and approved by institutional review board of the University of Hong Kong/Hospital Authority Hong Kong West Cluster (IRB HKU/HA HKWC, No. 09-377) and Kowloon West Cluster (KWC-REC No. KW/EX-13-013(59-14)).

## 3. Results

A total of 1522 colposcopies were performed in 1311 subjects during the study period from June 2010 to August 2017. Among the 1311 subjects, 160 had two colposcopies, 24 had three colposcopies, and 1 subject had four colposcopies during the study period due to persistent abnormal smear or persistent positive HPV results. The clinical information of these 1522 colposcopies is shown in Table 1. Among the cases with colposcopic-directed biopsies, 84.1% had up to two biopsies, while 15.9% had three or more biopsies. All the procedures were performed in the outpatient setting in our colposcopy clinic. There were no complications observed during the study period.

There were 118 high-grade cervical intra-epithelial neoplasia (CIN2 and CIN3) diagnosed during the study period. There was no invasive disease identified. The colposcopic impression of these 118 high-grade CIN cases is shown in Table 2. 

Overall, colposcopic-directed biopsy detected 50.8% (60 cases) of the 118 high-grade CIN cases, while four-quadrant biopsy detected 86.4% (102 cases) (*p* < 0.0001) (Table 3). Sixteen cases of high-grade CIN were not diagnosed using four-quadrant cervical biopsy. Among these 16 cases, 9 cases (7.6%) were detected using colposcopic-directed biopsy, and 7 cases (5.9%) were detected using endocervical curettage. 

**Figure 1:**
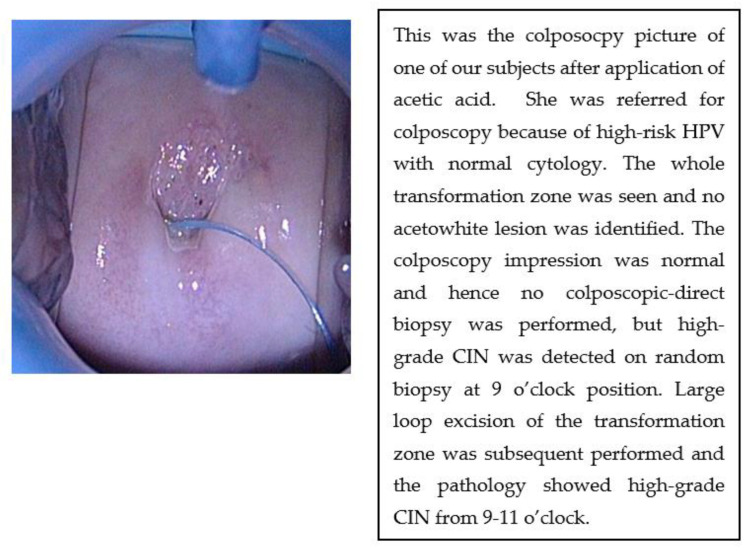
Colposcopic picture after application of acetic acid in one of the subjects.

Twenty-seven cases (22.9%) of high-grade CIN were diagnosed in patients with normal or unsatisfactory colposcopy. An impression of high-grade colposcopy findings was only made in 27 cases (22.9%). Among the remaining 64 cases with low-grade colposcopic impressions, colposcopic-directed biopsy only detected 36 cases (56.3%), while four-quadrant biopsy detected 53 cases (82.8%) (*p* = 0.0011). An example of a colposcopic picture is shown in Figure 1.

In the overall study population, the yield of high-grade CIN from four-quadrant cervical biopsy was 6.7% compared with only 3.9% from colposcopic-directed biopsy (*p* = 0.0006). Among the 1522 colposcopy cases, 90 cases were classified to have high-grade colposcopic impression. There was no difference in the yield of high-grade CIN from the two biopsy methods in this group. In the remaining 1432 colposcopies with low-grade/normal/unsatisfactory findings, the yield of high-grade CIN from four-quadrant cervical biopsy was significantly higher than that from colposcopic-directed biopsy (5.4% vs. 2.5%, *p* = 0.0001) (Table 4).

There were 67 high-grade CIN in the group of ASCUS/LSIL/ASC-H smears (541 subjects). Among the 67 cases, only 34 cases (50.8%) were detected using colposcopic-directed biopsy. An additional 30 cases (44.8%) were diagnosed using four-quadrant cervical biopsy, and 3 cases were only detected using endocervical curettage. The yield of high-grade CIN from four-quadrant cervical biopsy in subjects with high-grade smears (HSIL/ASC-H), low-grade smears (ASCUS/LSIL) and normal smears was 51.2%, 9.5% and 3.3%, respectively.

Human papillomavirus (HPV) status was available in 1346 cases, in which 1011 cases were found to have high-risk HPV in cervical samples before colposcopy. The percentage of high-risk HPV in ASCUS smears, LSIL smears, ASC-H smears and HSIL smears were 96.7%, 73.2%, 92.9% and 100%, respectively. Among these 1011 cases with high-risk HPV, there were 91 cases of high-grade CIN. Colposcopic-directed biopsy detected 46 cases (50.5%), while four-quadrant cervical biopsy detected 78 cases (85.7%) (*p* < 0.0001). The yield of high-grade CIN from four-quadrant cervical biopsy in this group was 7.7%. 

Endocervical curettage was performed in 1488 colposcopic examinations, and 22 cases of high-grade CIN were detected. Out of these 22 cases, 7 cases of high-grade CIN were detected using endocervical curettage in subjects without high-grade CIN on colposcopic-directed biopsies or four-quadrant biopsies. Among these seven subjects, two of them had normal findings at colposcopy, four had low-grade impressions, and one had a colposcopic impression suggestive of high-grade changes.

For glandular lesions, six cases of glandular dysplasia and four cases of adenocarcinoma in situ (AIS) were diagnosed during the study period. All the glandular lesions except one AIS were detected using four-quadrant biopsy, while colposcopic-directed biopsy only diagnosed two cases of glandular dysplasia and one case of AIS. The sensitivity of detecting significant glandular lesions was 30% using colposcopic-directed biopsy and 90% using four-quadrant biopsy. The remaining one case of AIS was diagnosed using endocervical curettage in a subject with normal colposcopic-directed biopsy and four-quadrant biopsy.

## 4. Discussion

In our study, only half of the high-grade CIN (60 out of 118 cases) were diagnosed using colposcopic-directed biopsy, and more than one-fifth of the high-grade CIN (27 cases, 22.9%) were diagnosed using four-quadrant cervical biopsy in women with normal or unsatisfactory colposcopy. We found that a high-grade colposcopy finding was quite reliable in directing the worst area for biopsy but not in patients with low-grade or normal/unsatisfactory colposcopy.

The reported sensitivity of colposcopy in detecting high-grade CIN varied widely (29–93%) in the literature [14,15]. The large variation in sensitivity could be due to the inherent limitations of colposcopy in detecting small lesions, especially in patients with difficult examinations due to vaginal atrophy or a retracted transformation zone. The variation can also be explained by the difference in the technique of colposcopy and the number of cervical biopsies taken during each colposcopy. In a questionnaire survey of 749 accredited colposcopists, only 45.8% of the respondents reported taking biopsies for most cases. When performing colposcopic-directed biopsies, 84.2% of the respondents aimed to obtain up to two samples, whereas 15.8% aimed for at least three or more samples [16]. The biopsy rate reported in a retrospective study on colposcopy practice in the US was much lower, with only 1.47 biopsies/patient for high-grade referrals and 0.97 for low-grade referrals [4]. In our study, 84.1% of the cases had up to two biopsies taken, while 15.9% had three or more samples. This was very similar to the findings in the questionnaire survey. In view of the variable sensitivity of colposcopy, the use of random cervical biopsy during colposcopy in different clinical scenarios has been evaluated by a number of studies in the past two decades. 

### 4.1. Role of Four-Quadrant Biopsy in Women with Positive Colposcopy 

In our study, 91 cases of high-grade CIN were diagnosed in the group with positive colposcopy. Colposcopic-directed biopsy of the abnormal area only detected 60 out of the 91 cases (65.9%), with another 26 cases (28.6%) diagnosed using four-quadrant cervical biopsy and the remaining 5 cases (5.5%) from endocervical curettage. Pretorius et al. reported an increase in the detection rate of CIN2 or worse from 57.1% to 94.5% by adding random cervical biopsies at the squamocolumnar junction in the quadrants that did not have lesions. The yield of CIN2 or above for random biopsy was 17.6% when the cytology was high grade and 2.8% with low-grade cytology [11]. Another study published by the same group in 2011 showed that 25.7% of CIN3 or above in the series were detected using random biopsy at the cervical quadrants without visible lesions [15]. Among the 31 invasive cancers in this series, only 27 cases were diagnosed using colposcopic-directed biopsy, with another 3 cases detected using random biopsy at the negative quadrant, and another 1 case diagnosed using endocervical curettage. The result suggests that up to 10% of invasive cancer would have been missed if only colposcopic-directed biopsy was performed. The usefulness of random biopsy in women with positive colposcopy was challenged by Song et al. They reported that the yield of high-grade CIN on random biopsy in the negative quadrant in women with positive colposcopy was less than 4%, and they, therefore, concluded that random biopsy in the negative quadrant was not effective in detecting high-grade CIN in women with positive colposcopy [17]. However, the overall incidence of high-grade CIN in their study was only 4.3%, which was much lower than the reported incidence in most of the published literature [7]. One possible reason for the low incidence of high-grade CIN in this study was that 74% of colposcopies were performed in patients with a normal smear, of which only 10% had high-risk HPV. Normal cytology with negative high-risk HPV was not the usual indication for colposcopy. This exceptionally low rate of high-grade CIN might account for the low yield on random biopsy in this study.

### 4.2. Role of Four-Quadrant Biopsy in Women with Negative Colposcopy 

In our study, more than one-fifth of the high-grade CIN (27 cases, 22.9%) were diagnosed using four-quadrant cervical biopsy in women with normal or unsatisfactory colposcopy. The role of random cervical biopsies in women with negative colposcopy has also been investigated by several studies. The post hoc analysis of the ATHENA trial reported that 20.9% of all the CIN2 or worse histology was diagnosed using a single random biopsy in women with negative colposcopy. This was very similar to the findings in our study, in which 22.9% of all the high-grade CIN was diagnosed using four-quadrant biopsy with ECC in women with negative or unsatisfactory colposcopy [9]. A pooled analysis of 3213 women with abnormal screening tests (abnormal cytology or positive high-risk HPV) but negative colposcopy from 17 population-based studies in China showed that the detection rate of random four-quadrant cervical biopsies in women with negative colposcopy was 4.3% for CIN2, 2.6% for CIN3 and 0.2% for invasive cancer [18]. A recent study of 173 women with low-grade cervical cytology (ASCUS or LSIL) and a normal colposcopy in Denmark showed that 22% of the women were diagnosed with high-grade CIN when four-quadrant random cervical biopsies were performed. The detection of high-grade CIN increased from 11% when only one biopsy was used to 22% when four biopsies were used [19]. The yield of high-grade CIN from four-quadrant cervical biopsies in women with low-grade smear was lower in our study at 9.5%. One possible reason is the difference in the percentage of high-risk HPV between these two studies. Most of the women included in the Danish study had high-risk HPV (87.2% for ASCUS smears and 100% for LSIL smears), whereas high-risk HPV was found in only 73.2% of LSIL cytology in our study.

### 4.3. Role of Endocervical Curettage

In our study, the yield of high-grade CIN using endocervical curettage alone was 1.5% (22 cases out of 1499 endocervical curettage). In total, 7 (5.9%) out of the 118 cases of high-grade CIN were detected on endocervical curettage in subjects without high-grade CIN on colposcopic-directed biopsy or four-quadrant random biopsies. The additional yield of high-grade CIN from endocervical curettage after colposcopic-directed biopsy and four-quadrant biopsy was only 0.5% in our study. The additional yield of endocervical curettage in detecting high-grade CIN varied greatly in the literature, depending on the indication of the colposcopy and the number of cervical biopsies taken. A study by Van Der Marel et al. showed that the yield of high-grade CIN using ECC was 11.9%; however, all these high-grade CIN were found in women with high-grade cytology [20]. Liu et al. reported that the overall detection of high-grade CIN using endocervical curettage was 14.4%, but endocervical curettage found only 3.9% additional high-grade CIN in women with up to four colposcopic-directed biopsies [21]. The American Society for Colposcopy and Cervical Pathology (ASCCP) recommended endocervical curettage for patients with high-grade cytology, human papillomavirus 16/18 infection, positive p16/Ki67 dual staining, for those previously treated for cervical pre-cancer or considering an observation of CIN2 and when the squamocolumnar junction is not fully visualized at colposcopy [22]. Our study shows a very low additional yield of high-grade CIN (0.5%) using endocervical curettage after colposcopic-directed biopsy and four-quadrant random biopsy. Our data suggested that endocervical curettage may be omitted if colposcopic-directed biopsy and four-quadrant cervical biopsy were performed.

### 4.4. Role of Colposcopic-Directed Biopsy

Our study shows that 86.4% of the high-grade CIN were diagnosed using four-quadrant cervical biopsy, and an additional 5.9% were diagnosed using endocervical curettage. In other words, performing colposcopic-directed biopsy only added less than 8% to the total number of high-grade CIN after 4-quadrant biopsy and endocervical curettage Colposcopy examinations require special equipment (i.e., a colposcope) and training. In many parts of the world, examinations are performed in a dedicated clinic with trained personnel (doctors or nurses). Depending on the healthcare system, it is not uncommon for women to have to wait for a few months before they can be seen in a colposcopy clinic. For example, the standard waiting time for colposcopy for low-grade cytology or positive high-risk HPV in the United Kingdom is 18 weeks. It has been well reported that abnormal cytology or HPV results cause a negative psychological impact on these women [23,24,25,26], and waiting for the initial tests or subsequent procedures was identified as distressing [25]. Our results show that performing four-quadrant cervical biopsy and endocervical curettage alone (without colposcopic-directed biopsy) could already diagnose more than 90% of high-grade CIN cases. This approach would allow women to have further investigation in their gynecologist office without the need to wait for a few months before being seen in a colposcopy clinic. This might help to relieve women’s anxiety while waiting for a colposcopy, and it might also improve the default rate for further investigation after abnormal cytology or HPV tests. However, this approach would involve the pathological examination of a larger number of biopsy specimens. The cost-effectiveness of this approach would depend on the setting of the healthcare system and the prevalence of high-grade CIN in the population. Further research should be carried out to investigate if this would be a cost-effective model in different healthcare settings.

Our findings suggest that a high-grade colposcopy finding was quite reliable in directing the worst area for biopsy but not in patients with low-grade or normal/unsatisfactory colposcopy. In our study, half of the high-grade CIN would be missed in the group with ASCUS/LSIL/ASC-H smears if only colposcopic-directed biopsy was performed. This was an important finding for the management of these patients. This was especially important in the management of women with low-grade cytology. In patients with HSIL cytology, most international guidelines recommend a diagnostic excisional procedure to exclude underlying high-grade CIN, even in patients with normal or low-grade colposcopy findings and cervical biopsy [27]. However, this was not the case in patients with low-grade histology. These patients would be advised to return to cytology/HPV surveillance. The default rate after colposcopy assessment varies in different socioeconomic and geographical backgrounds [28,29], but a negative or low-grade colposcopy and biopsy might give a false sense of reassurance in some of the patients who might have an underlying high-grade CIN which was not detected using colposcopic-directed biopsy alone. 

One postulation for our finding was that if the HSIL area was too small, it might not appear as acetowhite or an iodine-negative lesion on colposcopy, and as a result, it was missed during the examination. One might argue that these smaller lesions detected on random biopsy only might have a different natural course and clinical significance compared with the biopsy obtained from the abnormal area during colposcopy. We were unable to evaluate this in our study, and we were not aware of any literature on this area. Therefore, these patients with high-grade CIN detected on random biopsy should be managed according to the current guidelines for high-grade CIN.

## 5. Conclusions

Our study shows that the overall sensitivity of colposcopy and directed biopsy in detecting high-grade CIN was only around 50%. High-grade colposcopy findings were quite reliable in directing the worst area for biopsy but not in patients with less obvious abnormalities. Therefore, four-quadrant cervical biopsies should be considered for all patients, especially those with low-grade/normal/unsatisfactory colposcopy.

## Figures and Tables

**Table 1:** Baseline demographics.

Age	No. of Patients (Percentage)
25–34	180 (11.8%)
35–44	558 (36.7%)
45–54	557 (36.6%)
≥55	227 (14.9%)
Menopausal Status	
Pre-menopausal	1161 (76.3%)
Post-menopausal	342 (22.5%)
Missing data	19 (1.2%)
Smoking	
Never smoker	1292 (84.9%)
Ex-smoker	84 (5.5%)
Current smoker	144 (9.5%)
Missing data	2 (0.1%)
Cervical smear	
Normal	966 (63.5%)
ASCUS	250 (16.4%)
LSIL	264 (17.3%)
ASC-H	27 (1.8%)
HSIL	14 (0.9%)
Missing	1 (<0.1%)
High-Risk HPV	
Positive	1011 (66.4%)
Negative	335 (22.0%)
Unknown	176 (11.5%)

**Table 2:** Colposcopic impression of the high-grade CIN cases.

Colposcopic Impression	No. of Cases (*n* = 118)
Unsatisfactory	10 (8.5%)
Normal	17 (14.4%)
HPV/LSIL	64 (54.2%)
HSIL	27 (22.9%)

**Table 3:** Detection rate of high-grade CIN from colposcopic-directed biopsy and 4-quadrant biopsy.

Colposcopic Impression	Number of High-Grade CIN	Directed Biopsy		Four-Quadrant Biopsy		*p* Value
		Number	Detection Rate	Number	Detection Rate	
Normal/Unsatisfactory	27 ^a^	0	0%	25	92.6%	-
Low grade	64	36	56.3%	53	82.8%	0.0011
High grade	27	24	88.9%	24	88.9%	1.0000
Total	118	60	50.8%	102	86.4%	<0.0001

^a^ Two of the twenty-seven high-grade CIN were detected using endocervical curettage.

**Table 4:** The yield of high-grade CIN from colposcopic-directed biopsy and 4-quadrant biopsy in the overall study population.

Colposcopic Impression	Number of Colposcopies	Directed Biopsy		Four-Quadrant Biopsy		*p*-Value
		Number	Yield of High-Grade CIN	Number	Yield of High-Grade CIN	
Normal/Unsatisfactory/Low-grade	1432	36	2.5%	78	5.4%	0.0001
High grade	90	24	26.7%	24	26.7%	1.0000
Total	1522	60	3.9%	102	6.7%	0.0006

## Data Availability

The data presented in this study are available on request from the corresponding author.
